# Resveratrol and Neuroprotection: Impact and Its Therapeutic Potential in Alzheimer's Disease

**DOI:** 10.3389/fphar.2020.619024

**Published:** 2020-12-30

**Authors:** Md. Habibur Rahman, Rokeya Akter, Tanima Bhattacharya, Mohamed M. Abdel-Daim, Saad Alkahtani, Mohammed W. Arafah, Norah S. Al-Johani, Norah M. Alhoshani, Nora Alkeraishan, Alhanof Alhenaky, Omar H. Abd‐Elkader, Hesham R El-Seedi, Deepak Kaushik, Vineet Mittal

**Affiliations:** ^1^ Department of Pharmacy, Southeast University, Banani, Dhaka, Bangladesh; ^2^ Department of Pharmacy, Jagannath University, Sadarghat, Dhaka, Bangladesh; ^3^ School of Chemistry and Chemical Engineering, Hubei University, Wuhan, China; ^4^ Department of Zoology, College of Science, King Saud University, Riyadh, Saudi Arabia; ^5^ Pharmacology Department, Faculty of Veterinary Medicine, Suez Canal University, Ismailia, Egypt; ^6^ Physics & Astronomy Department, Science College, King Saud University, Riyadh, Saudi Arabia; ^7^ International Research Center for Food Nutrition and Safety, Jiangsu University, Zhenjiang, China; ^8^ Pharmacognosy Group, Department of Pharmaceutical Biosciences, Uppsala University, Uppsala, Sweden; ^9^ Department of Pharmaceutical Sciences, Maharshi Dayanand University, Rohtak, India

**Keywords:** Alzheimer's disease, resveratrol, oxidative stress, neuroprotective, therapeutic agent, bioavailability

## Abstract

Alzheimer’s disease (AD) is a progressive cortex and hippocampal neurodegenerative disease which ultimately causes cognitively impaired decline in patients. The AD pathogen is a very complex process, including aggregation of Aβ (β-amyloid peptides), phosphorylation of tau-proteins, and chronic inflammation. Exactly, resveratrol, a polyphenol present in red wine, and many plants are indicated to show the neuroprotective effect on mechanisms mostly above. Resveratrol plays an important role in promotion of non-amyloidogenic cleavage of the amyloid precursor protein. It also enhances the clearance of amyloid beta-peptides and reduces the damage of neurons. Most experimental research on AD and resveratrol has been performed in many species, both *in vitro* and *in vivo*, during the last few years. Nevertheless, resveratrol’s effects are restricted by its bioavailability in the reservoir. Therefore, scientists have tried to improve its efficiency by using different methods. This review focuses on recent work done on the cell and animal cultures and also focuses on the neuroprotective molecular mechanisms of resveratrol. It also discusses about the therapeutic potential onto the treatment of AD.

## Introduction

Alzheimer's disease (AD) induces executive control and memory loss, a neurodegenerative disorder of the brain. It also affects long-term declarative memory and working memory of the patient. It correlates with the functional and structural parameters of the brain. It works on the pathology of formation of memories from the framework and molecular level of neural networks. Dementia also occurs in ageing stage due to the presence of Alzheimer's disease (Kelley and Petersen, 2007; Jahn, 2013). While AD was established more than 100 years ago, attempts are now being made to find new chemical products (i.e., natural antioxidants) that function at different points to avoid the progression of this disease. After studying of human experimental and accumulating data, it concludes that the pathogenesis of AD caused by the process of oxidative stress (OS) is produced from reactive oxygen species (ROS). This process takes place before appearing the molecular events (neurofibrillary tangles and β-amyloid plaques) and symptoms of AD. It also leads to damage of tissue by several pathways of various cellular molecules present into the neuron. The process of damage of cellular components like proteins, lipids, and nucleic acids (like RNA and DNA) caused by ROS leads to death of cells by apoptosis and necrosis process. After weakening of cellular antioxidant defense system, more damage of cells takes place. With the help of antioxidants, there can be prevention of tissue damage, and it also improves both neurological and survival outcomes (Gilgun-Sherki et al., 2003; Feng and Wang, 2012). The pathogenesis and development of AD are not well understood. However, neuronal loss, synaptic interconnections, and glial proliferation are widely studied, including neurotic plaques (senile plaques) (Selkoe, 2001). Thus, while the explanations for AD remain doubt, two commonly identified pathological features remain, senile plaque (SPs) and neurofibrillary tangles (NFTs). The SPs are the β-amyloid (Aβ), a dystrophic neuritis nucleus, activated by astrocytes, and microglia. NTFs consist of hyperphosphorylation and abnormal tau protein deposition. According to a study, it shows that by using resveratrol, we can easily slow down the effect of AD. The neuroprotective nature of resveratrol takes place by inhibiting Aβ (β-amyloid) aggregation, by exerting anti-inflammatory activity and by scavenging oxidants. The neuroprotective action of resveratrol in neuroblastoma cells of human, exposed to Aβ-metal complex or Aβ (Granzotto and Zatta, 2011). For a long time, scientists have sought to diagnose and mitigate cognitive conditions. Unfortunately, there has not been any reversal of current deficits or disease progression treatment at present. In another study, results show that when patients of AD are treated with omega-3 fatty acid docosahexaenoic acid (DHA), the symptoms of AD seem moderate to mild in patients (Yaffe, 2010). Many other treatments with several drugs have been widely used to alleviate symptoms of AD, including some inhibitors of cholinesterase and, in the meantime, a glutamate antagonist N-methyl D-aspartate form (NMDA) receptor. However, the complex mechanisms have shown that these compounds produce a range of side effects and relatively small benefits (Allain et al., 2003). For instance, cholinesterase inhibitors can have temporary and modest effects on memory loss and motor function improvements, but side effects such as nausea and diarrhea are typically intolerable (Kim et al., 2010). In clinical trials, tacrine, another cholinesterase inhibitor, was reduced into low oral bioavailability and significant hepatotoxicity. In the meantime, a hoping drug has proven to have less clinical success than the cholinesterase inhibitors (Kim et al., 2010). Resveratrol is a natural compound obtained from plants that occurs primarily in grapevine and other fruit specie. Its diverse biological characteristics including antioxidant, anti-inflammatory, and neuroprotective activities attract wide attention. Resveratrol may indirectly activate the expression of SIRT1 and leads to AD neuroprotection (Beher et al., 2009). Resveratrol (3, 5, 4′-trihydroxy-trans-stilbene) is a kind of polyphenol produced in several plants, especially from grapes skin and seeds, and acts as a phytoalexin against pathogens like bacteria or fungi (Li et al., 2012). Resveratrol has demonstrated diverse biological activities in the treatment of cardiovascular and cancer, as well as revenant disorders, including brain diseases and AD, and produces protective effects such as antioxidants, antimicrobial, phytoestrogenic, vasorellaxant, cardioprotection, and anticancer (Baur et al., 2006; Holland et al., 2008; Oomen et al., 2009). Many studies were carried out to find out whether resveratrol can take on the therapeutic potential for AD and other neurodegenerative diseases. However, the long-term effects of human supplementation are still under investigation, including resveratrol and AD. The studies show that resveratrol has taken part in many pathophysiological AD courses (Baur and Sinclair, 2006). Recent studies on cell cultures, animal models, and primarily on the molecular mechanisms of neuroprotective effects of resveratrol are explored in this review, and the therapeutic potential of the disease is investigated. We also address the role of resveratrol in AD therapy, and it also discusses the possible benefits of resveratrol as a medical agent known as anti-AD.

### Specific Pathogens and Various Treatment Targets

**FIGURE 1:**
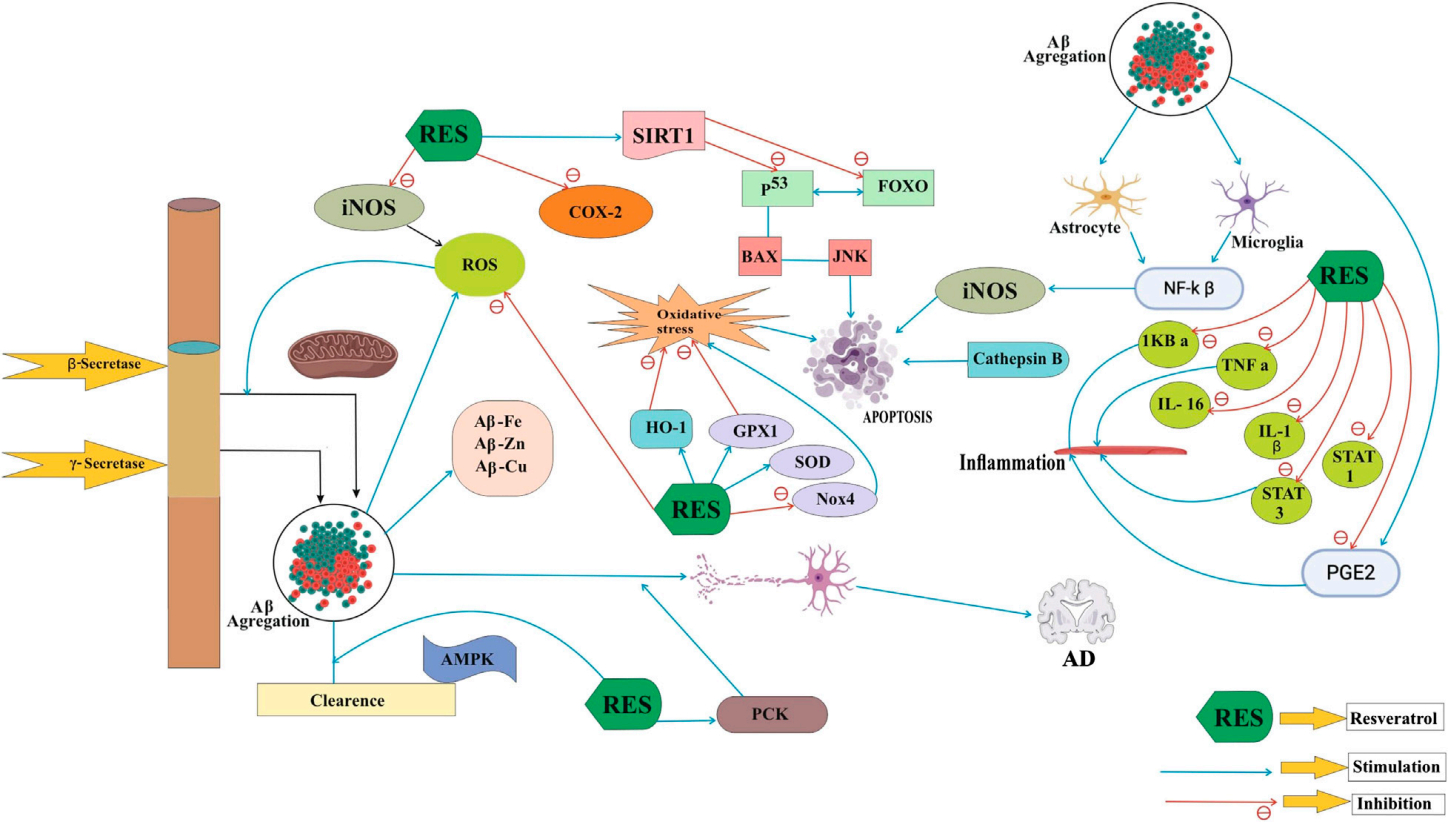
Neuroprotective functions of resveratrol in AD pathogenesis.

The exact mechanism of AD is unclear, but the compound resveratrol shows antioxidant activity on SIRT1 (silent information regulator-1) and it also causes neuronal differentiation. The growth of neurons highly dependent upon SIRT1 that is also responsible for prevention of apoptosis death in neurons. Pathogenic mechanisms of AD are very complex; it includes Aβ accumulation, oxidative stress, tau protein phosphorylation, and inflammation (Figure 1). In this case, most therapies cannot consider every aspect. AD describes pathological properties, especially neuritis and NFTs that are visible in AD patients' brains by microscopy (Perl, 2010). The presence of Aβ (β-amyloid peptides) is widely responsible for learning and memory ability of AD. By inhibiting the process of Aβ peptide aggregation, we can easily treat the disease of AD. Neurotic plaques are Aβ peptide extracellular insoluble deposits, which are also identified as senile plaques. In 1992, an amyloid discovery was concluded that Aβ accumulation was the key cause of AD, and the hypothesis has since been gradually recognized (Hardy, 2006). Amyloid precursor protein (APP) is abnormally cleavage by β- and γ-secretase (not α-secretase), leading to an unnecessary cortex–hippocampal extracellular Aβ accumulation in brains. The buildup of Aβ contributes to progressive neuronal failure, neural circuit interdiction, and AD neurological characteristic deterioration. Several investigations occur with AD therapies, along with clinical trials that block pathogenic amyloid-β peptides. It also rescues vulnerable neurons from the process of degeneration (Roberson and Mucke, 2006). However, APP is separated by the α-secretase pathway into soluble amyloid α precursor α (sAPPα), which may reduce the generation of Aβ and protect the neurons relatively by fostering axon growth. Consequently, the principal goals of AD products are β- and gamma-secretase, but α-secretase improvement can also have the same anti-AD therapy effect.

Oxidative stress has been particularly affected by the pathogenesis of neurodegenerative diseases such as AD and has become extremely susceptible to oxidative damage because of the greater degree of polyunsaturated grain and the relative lack of antioxidant role in other organisms. As we can see, in case of Alzheimer’s disease (AD), there is an increasing level of proteins, oxidized nucleic acid, and lipids. It is quite resemblance with Down’s syndrome in young adults. The process of cellular damage and oxidative stress (OS) takes place in early stage of AD. Several anti-inflammatory agent, antioxidants, mitochondrial protective agents, and metal chelators are used against OS therapies for AD. Some of them show neuroprotective effect against animal and cellular models of AD (Nunomura, 2013). The generation of damaged mitochondria during oxidative stress, primarily by nitrogen oxide synthase (iNOS) and cyclooxygenase-2, leads to injury, loss of membrane integrity, and a loss of lipid peroxidation and nuclear acids. ROS increasing Aβ production, and Aβ inducing oxidative stress, a vicious circle between ROS and Aβ accumulation, may accelerate the progression of AD (Li et al., 2012). The ROS increases AD development, Aβ growth, and the oxidative stress caused by Aβ, a brutal process between the accumulation of ROS and Aβ (Li et al., 2012). Resveratrol is used to not only minimize iNOS concentrations and lipid peroxidation in neuronal cells as an antioxidant but also increase hemoxidation production-1 (HO-1) to prevent oxidation. Neuroinflammation recently showed that it contributes significantly to AD pathogenesis (McGeer and McGeer, 2003). Flammative changes in the AD brain, particularly in the amyloid deposits, including microglia activation, astrocytes, and macrophages, can be observed. The changes like neuroinflammation observed in the several affected areas of the brain combines with epidemiological evidence that produces protective effect of anti-inflammatory agents which may play an important role in treating AD (McGeer and McGeer, 2003). Many data in cell and mouse cultures indicate that aggregated Aβ is responsible for activating and releasing astrocytes and microglia, leading to the release of significant quantities of inflammatory mediators including cytokines, free radicals, and nitric oxide (NO)(Capiralla et al., 2012; Tansey et al., 2007; Velez-Pardo et al, 2002). Aβ triggers the respiratory burst of microglia and produces ROS and tumor necrosis factor-alpha (TNF-α), which aggravates Aβ deposition and further neuronal dysfunction and eventual death (Holtzman, 2008; Wilcock et al., 2009). Synaptic dysfunction takes place during the early stage of AD, and synapse failures are found during the later stage. The hippocampus and stroke impede excitatory transmission, leading to loss of memory (Selkoe, 2002; Mallucci et al., 2007). While Aβ deposition can lead to neuronal loss, apoptosis is the principal method of neuronal synapse loss. Multiple factors, such as stress, glucose metabolism, and mitochondrial excitotoxic damage, may trigger apoptosis in AD models. In the apoptosis cycle of AD, other substrates such as p53, FOXO, and ROS are involved. There seems to be a possible treatment of AD for apoptosis downregulation.

Tau protein is a highly soluble, microtubule-associated protein (MAP). Some of these protectors reside in central and peripheral neurons and act as stabilizers of microtubules. Deposition of tau hyperphosphorylation and amyloid-β is a cardinal pathologic feature of AD which leads to the formation of neurofibrillary tangles and neuronal plaques. Herbal compound like resveratrol is highly responsible for anti-AD effect into the brain (Villaflores et al., 2012). Tau is a phosphoprotein that is regulated by several kinases in the phosphorylation of tau. If the tau protein has a phosphorylated material, it derives abnormally, like AD, from microtubules and aggregates (Alonso et al., 2001). The phosphorylated tau protein disassembles neurofibrillary engravings in microtube and aggregates, the distinctive characteristic of AD pathology. When tau is polymerized as neurofibrillary tangles, it loses its connection to the tubulin and the microtubules. Hence, inhibition of tau pathological hyperphosphorylation may be a therapeutic target for AD as well as other disorders (Alonso et al., 2008; Iqbal et al., 2010). The therapies for many incremental neurodegenerative disorders, such as Huntington disease, Parkinson's disease, and AD, were neurological problems. Resveratrol has strong metabolic effects and is considered an example of caloric limitation (Pasinetti et al., 2011). Resveratrol has shown neuroprotective effects on pleiotropic functions in recent years. In general, studies demonstrate that in different models, both *in vitro* and *in vivo*, resveratrol has significant neuroprotective properties (Saiko et al., 2008). According to Kores *et al*, it is believed that resveratrol is an important constituent to cure neurological disorder like Alzheimer’s disease and cardiovascular disorders. By using the molecular docking approach, it is concluded that new potential were found to targets of resveratrol. The results concluded that more than three human proteins are already known to bind with resveratrol. A large number of protein were discovered by this docking process which connects to resveratrol. It leads to a new possibility to cure a variety of disease like AD (Kores et al., 2019). According to Rong Ma *et al*, the diseases such as Alzheimer’s disease (AD) and diabetes mellitus (DM) mostly coexist in the patients. Due to the conditions of one disease, there is an increasing risk of another one. Both diseases have the same pathophysiological mechanism, oxidative stress, inflammatory signaling pathways, and cell apoptosis. The level of Aβ also similarly elevates into the brain. By using compound resveratrol, both diseases DM and AD can be treated simultaneously by modulating oxidative stress and by reducing inflammation into the brain (Ma et al., 2019). According to Chen *et al*, resveratrol shows positive results in animal models of AD. There are a number of theories on the mechanism of action of resveratrol. By using rodent AD models, the efficacy of resveratrol was studied. The analysis of results shows that resveratrol is highly effective as a neuroprotective agent. It includes some clinical trial on the studies of treatment of AD (Chen et al., 2019). According to Yan *et al*, due to ageing of population, the number of cases of AD increases in the society. Various phytochemicals have been used for the treatment of AD, from which resveratrol is one of them. There is a possible relationship between AD and factors like glutamine receptors (AMPARs), synapses, estrogens, and silent information regulator 1 (SIRT1). Treatment of AD with resveratrol compound is highly demanding among all of them (Yan et al., 2020).

### Resveratrol: Neuroprotective Effects *in vivo*

In animal models, particularly in rat models, resveratrol was found to be neuroprotective (Table 1). The routine moderate consumption of red wine reduces amyloid-AD neuropathology substantially and mitigates the Aβ-associated mouse memory loss Tg2576 (Wang et al., 2006). Recent studies about Tg2576 mice indicated that extracellular accumulation of soluble Aβ oligomers was largely responsible for AD dementia and memory deficits (Cleary et al., 2005). Treatment with polyphenolic compounds from grape seed extract (GPSE) decreased Aβ peptide oligomerization and decreased amyloid Tg2576 mice’s cognitive loss (Wang et al., 2008). In the treatment of resveratrol APP/PS1 mice, the amount of activated microglia decreases considerably and indicates that resveratrol decreases in part inflammation, regardless of its effects on amyloid deposition (Jeon et al., 2012). Studies in a C57Bl/6J mice model have also shown that the number of rears in high-grade mice treated with resveratrol has decreased substantially in ambulatory locomotives operation and is likely to decrease ((Lagouge et al., 2006). The findings showed that the resveratrol effect on both muscle and brown adipose tissues could increase mitochondrial activity, resulting in greater consumption of energy, greater aerobic efficiency, and increased sensorimotor function (Lagouge et al., 2006). Sharma and Gupta indicated that ICV STZ group rats chronically treated with trans-resveratrol showed significantly increased retention latencies and shorter transfer latencies on the elevated plus-maze, but no significant difference in the locomotor activity of sham. A study was conducted on the intracerebroventricular (ICV) streptozotocin (STZ) model of various types of rats having dementia of Alzheimer’s type. The compound resveratrol produces effect on ICV STZ in rats. These were treated with 10 and 20 mg/kg dose of resveratrol for 21 days. A rising of brain glutathione and an increasing level of MDA (malondialdehyde) in the brain treated with ICV STZ is observed in rats. So it concludes that it is possible to cure neurodegenerative disease like AD with resveratrol (Sharma and Gupta, 2002). Some rat models used colchicine in ICV (15 μg/5μl) that can cause cognitive impairment. The level of malondialdehyde (MDA) and nitrite decreased the activity of glutathione, acetylcholinesterase improved, and colchicine cognitive impairment increased significantly for 25 days (Kumar et al., 2007). Additionally, resveratrol therapies in diabetic rats may avoid increased AChE behaviors in cholinergic neurotransmission, which can then increase cognitive dysfunction, and boost cognition (Schmatz et al., 2009). In the analysis, brain-derived neurotrophic factor (BDNF) mRNA of the hippocampal Sprague–Dawley rat model was stimulated by oral resveratrol. The BDNF has similar functions such as resveratrol, and the study has shown that resveratrol can support BDNF expression (Rahvar et al., 2011). The SIR2 genes promote the reproduction of many animals and the benefits for the well-being of calorie reduction of SIR2 genes. SIRT1, one of seven NAD + mammals, has recently been shown to aid cellular regulation and to participate in many SIRT1 signal pathways (Bonda et al., 2011). The resveratrol ICV injection used the transgenic p25 mouse, AD pattern, and tauopathies to activate the SIRT1. In vehicle-treated animals, the cell death and neurodegeneration were evident after five weeks of induction of P25, but resveratrol administration was effective in reducing CA1 and CA3 hippocampal neurodegeneration by lower apoptotic marker–activated caspase-3 markers and the glial fibrillary acid (GFAP) (Kumar et al., 2007). P25-green fluorescent protein that expresses neurons showed that p25 can withstand and thrive on the hippocampus of resveratrol-treated rats. After 3 weeks of resveratrol therapy, learning capacity improved. This means that resveratrol offers neuroprotection and prevents animal models from slipping in cognitive stages (Kim et al., 2007a). In animal neurodegenerative disorders triggered by certain neurotoxicity, resveratrol is useful. The antioxidants and anti-inflammatory properties of the possible pathways can be responsible. Resveratrol pretreatment significantly reduces oxidative stress injury and improves motor and cognitive injury (Kumar et al., 2006; Zhang et al., 2010).

**TABLE 1:** Neuroprotective effects of resveratrol *in vivo*.

Animal model	Administration category	Effects	Reference
C57Bl/6 mice	Resveratrol in food	(1) Increasing microvascular density and lower vacuolar anomalies. (2) Improved efficiency in spatial orientation and memory.	Oomen et al. (2009)
Tg2576 mice	Cabernet Sauvignon to drink	Reduced amyloid neuropathology and loss of visual memory.	Wang et al. (2006)
C67BL/6J mice	Trans-resveratrol diet every day	Serum TNF-α decreased and cognitive function increased.	Jeon et al. (2012)
Tg2576 mice	Feeds on GPSE	Decreased Aβ peptide oligomerization and decreased cognitive deficits.	Wang et al. (2008)
APP/PS1 mice	Resveratrol diet	Decreased the amount of microglia activated.	Capiralla et al. (2012)
C57Bl/6J mice	Food resveratrol	Improved mitochondrial activity, aeroplane ability increased, and sensorimotors strengthened.	Lagouge et al. (2006)
Male mice, 2 months old.	i.p. injection of resveratrol	The AMPK in the brain is activated.	Dasgupta and Milbrandt (2007)
Wistar Councils, i.p. STZ injector	i.p. injection of resveratrol	This makes cholinergic neurotransmission easier for users to understand.	Schmatz et al. (2009)
Consequences, ICV colchicine administration	Chronic resveratrol treatment (P.O.)	The level of MDA decreased but the activity of GSH and AchE recovered.	Kumar et al. (2007)
Sprague–Dawley rat	Oral resveratrol	The effects of resveratrol can be positive on BDNF expression.	Banerjee et al. (2008)
Inducible p25 transgenic mice	Resveratrol ICV injection	Reduces hippocampal neurodegeneration and prevents cognitive deterioration.	Kim et al. (2007a)

### Resveratrol: Neuroprotective Effects *In Vitro*

For several *in vitro* models as a rat or human cells, neuroprotective effects of resveratrol have been observed (Table 2). In 10-day Sprague–Dawley rat pups, glutamate easily triggered the production of monocyte chemical protein1 (MCP-1). Resveratrol downregulated glutamate-induced extracellular signal-regulated kinase (ERK) activation and then resulted in decreased interleukin-1β (IL-1β) expression and the subsequent downregulation of MCP-1 in the hippocampus (Lee et al., 2010). There was a decrease in 1α-receptor-gamma-coactivator-(PGC-) proliferator-activated peroxisome in rats that indicated an increase in SIRT1 following primary resveratrol therapy. Higher SIRT1 levels were also correlated with protective functions of neurotoxicity of p25 or mutant SOD1 in the primary neurons. Resveratrol assisted neuroprotection and avoided cognitive decline with deacetylation of p53 and thereby reduced p53, an important cell death mediator (Kim et al., 2007a). The inhibition of intracellular calcium and ROS output has been found to prevent neuronal death caused by the NMDA (Ban et al., 2008). Aβ25–35 was taken to determine neuronal cell death from Aβ due to their similar toxic action mechanism in a study of primary hippocampal cultured cells. Dose-mediated cell death dependent on Aβ 25–35 decreases pretreatment resveratrol (15–40 μM) substantially, with a median effect of 25 μM, on hippocampal neuronal cells. In the meantime, treatment with resveratrol and posture had similar neuroprotective effects, although the findings were slightly lower in severity (Han et al., 2004). Protein kinase C (PKC), with a median effect of 20–30 μM and based on dosage, was phosphorylated by the resveratrol induce. The effects of resveratrol on PKC-α/βII, PCC-μ (Ser 916), and PKC-Ther were also substantially reduced. This indicates that the neuroprotection effects of P K-D (Thr505) affect resveratrol. In short, the PKC pathway had a significant role in the neuronal hippocampal toxicity of resveratrol (Han et al., 2004). NO donor SNP (100 μM) therapy in mixed cells (glial) and neuronal cells resulted in resveratrol and cell survival damage (5 ± 25 μM) and the median concentration of 5 μM and the average concentration (25 μM) investigated this survival gain. Resveratrol and other polyphenols from red wine were activated by possible antioxidant activities to save hippocampal cells from NO toxicity. Note: the effects on intracellular enzymes including COX/LOX, NOS, and PKC were not included because quercetin did not include this experiment. However, in lipopolysaccharide-activated macrophages, resveratrol (10 μM) was found to enhance NO generation and the suppression of iNOS. Different factors such as cell types and medicaments can lead to it (Bastianetto et al., 2000). Multi-accident regulation in COX/PGE2 can also inhibit the free formation and free radical formation of prostaglandin E2 (PGE2) in the primary microglial cultivated cells by active microglia cells. The LPS-media prostaglandin E synthase-1 (mPGES-1) and COX-1 expression can be reduced, but not COX-2 (Candelario-Jalil et al., 2007). Aβ has contributed to time-dependent cell growth in cultured rat astroglioma C6, but resveratrol pretreatment protects cellulose against Aβ toxicity. In response to Aβ in these experiments, resveratrol inhibited concentration-dependent NO-output and iNOS expression. Besides, the inhibitory role of Aβ aggregation of COX-2 in PGE2 C6 cells is shown in resveratrol. Alternatively, the Aβ translocation of NF-κB was disrupted by resveratrol before treatment (Kim et al., 2006).

**TABLE 2:** Neuroprotective effects of resveratrol *in vitro.*

Types of cell	Exposure	Effects of resveratrol	Reference
Primary cortical neurons	Exposure to NMDA	Hampered the intracellular calcium elevation and ROS output.	Ban et al. (2008)
Primary hippocampal cells	Aβ _25–35_ induced	Reduced cell death by Aβ 25–35, and phosphorylation was reduced by PKC-β.	Han et al. (2004)
Rat cortical primary neurons	Ionomycin and H_2_O_2_ diagnosis	Increased development of SIRT1, and cognitive decrease prevention.	Kim et al. (2007a)
Mixed (glial/neuronal) hippocampal cells	Treated with SNP or SIN-1	Rescued hippocampal cells against NO-induced toxicity and inhibited NO generation and suppressed iNOS in LPS-activated macrophages.	Bastianetto et al. (2000)
Hippocampal cells of the murine HT22 and primary cells of the hippocampal	Aβ, MEL and resveratrol are treated	MEL and resveratrol had inhibited activation of ERK, decreased development of ROS, rescued GSH, and reduced neuronal cell death. The synergistic effect of co-treatment.	Kwon et al. (2010)
Rat astroglioma C6 cells	Aβ is classified	Reduced production of NO and iNOS expression, inhibited PGE2accumulation, decreased the COX-2 expression, and prevented NF-μB from being translocated.	Kim et al. (2006)
PC12 cells	Aβ is classified	Aβ-soluble oligomers and conformers of the fibrillar remodels into large, nontoxic aggregates.	Ladiwala et al. (2010)
SK-N-SH cells	IL-1β stimulated	Reduced development of PGE2 and PGD2 by COX-2 reduction.	Ramesh et al. (2009)
HUVEC-derived EA.hy926 cells	DMNQ-induced	Reduced Nox4 expression, but enhanced SOD1 and GPx1 expression.	Spanier et al. (2009)

The RAW 264.7, BV-2, and the Ba/F3 pro-B cell line, the murine microglial cells, have been recently studied. The treatment of resveratrol was found to be substantially reduced in LPS-started RAW 264.7, and BV-2 cells including IL-6, M-CSF, CD54, IL-1ra, and TNF-α. All of them were regulated with NF-SB transcriptionally. The NF-SB reporting regulates the expression of the toxic factors of apoptosis iNOS and c cathepsin B. In RAW 264.7 and BV-2 the phosphorylated levels in IKKα, Iα, and NF-α decreased, while activation in Toll-like receptor (TLR) 4 was significantly inhibited by the phosphorylation of the Act, MyD88, and mediated kinase. On the other hand, resveratrol inhibited the STAT 1 and LPS activation of these two cell lines for signal transducers and transcription activators (STATs) and decreased dosage-dependent expression for iNOS or COX-2. Cytotoxicity-protected effect of resveratrol was shown to be Aβ 25–35 or Aβ 1–42 in cultured rat pheochromocytoma cells (PC12). In this sense, this phenolic compound displayed morphology and increased TUNEL positive cells, also being found to inhibit controlled, apoptotic cell death Aβ 25-35. It was reported to affect the Aβ 25–35 activated apoptotic signaling pathways. These include preventing PARP cleavage, recovering reduced Bcl-XL expression, preventing Bax protein levels, blocking JNK-like phosphate activation, and removing increased binding of NF-κB DNA (Jang and Surh, 2003). Resveratrol has also been found to dose-dependently restructure Aβ soluble oligomers and fibrillar conformers in rat PC12 cells (Ladiwala et al., 2010). Many candidates who were active in antioxidant AD and neuroprotection were observable melatonin (N-acetyl-5-methoxy-tryptamine, MEL). Co-treatments of MEL and resveratrol were more successful for Aβ 1–42 mediated neurotoxicity with the culturally cultivated murine hippocampal and primary hippocampal neuron cells. MEL and resveratrol have inhibited ERK activation, reduced ROS production, rescued glutathione (GSH) concentrations, and decreased neuronal cell mortality. A rise in gsk3β activities and AMPK activation induced by Aβ 1–42 were either inhibited by MEL or by resveratrol alone, and were synergistic with their co-treatment (Kwon et al., 2010).

Other liners of human cells are used to investigate the protective effects of resveratrol in AD (Table 2) also to rat cells as *in vitro* models*.* For example, the cell HEK293 was used to efficiency resveratrol on Aβ clearance, by using the cell line originally derived from human embryonic kidney cells cultivated with human APP695. Resveratrol had no impacts on α-, β-, or γ-medium APP-clips that did not influence the metabolism of the consumer and the development of resveratrol. Moreover, resveratrol was found in the sample. In HEK 293 cells, resveratrol failed, however, to promote intracellular proteasome-related clearing *via* Aβ without increasing the complete proteasome role by promoting the neutral endopeptidase (NEP), ECE-1, and ECE-2 by a β- or insulin-degrading enzyme (Marambaud et al., 2005). In addition, the antioxidant effect of the human umbilical vein endothelial cells (HUVEC), resveratrol regulated the gene expression of pro-oxidant and antioxidant enzymes. Incubation of human umbilical vein endothelial cells (HUVEC) and HUVEC‐derived EA.hy 926 cells with resveratrol resulted in a concentration‐ and time‐dependent downregulation of Nox4, the most abundant NADPH oxidase catalytic subunit (quantitative real‐time RT‐PCR). The same resveratrol regimen upregulated the mRNA expression of SOD1 and GPx1. Resveratrol has provided a new approach to reducing endothelial oxidative stress (Spanier et al., 2009). In human hippocampus slices, resveratrol can bind directly to both monomeric and fibrillar amyloid structures so that resveratrol can specifically stain Aβ plaques (Ge et al., 2012). Neuroblastoma cells SH-SY5Y were used to research the resveratrol inhibitory role of oligomeric cytotoxicity. Resveratrol has been shown to play a protective role in AD by eliminating the extension and disaggregating of Aβ42 fibrils, but without inhibiting the formation of Aβ42 oligomers (Feng et al., 2009). SH-SY5Y resveratrol activities in the metabolism of Aβ have been found in the presence or lack of resveratrol in human neuroblastoma cultures treated with Aβ complexes. Resveratrol was not directly anti-amyloidogenic and destabilizing fibril but played an important role in the neuroprotection of complexes of Aβ and Aβ-metals. Resveratrol, and its toxicity, scavenger Aβ-Fe and Aβ-Cu ROS generation after generation. Also, resveratrol effects have been studied in SK-N-SH cells in a human neuroblastoma cell line on the development of IL-1β–mediated prostanoids. Also at extremely low doses, resveratrol has been shown to reduce the development of PGE2 and PGD2 slowly. However, both mPGES-1 and COX-2 immune behavior, and COX-1, were not significantly diminished by the same dose of resveratrol. This means that resveratrol enhances the synthesis of the prostanoid without affecting the expression of COX-1, COX-2, or mPGE-1tion (Ramesh et al., 2009). Another research on the role of resveratrol in preventing oxidative stress was performed in SK-N-BE, another cell neuroblastoma line. Resveratrol was sirtinol-immune with antioxidant effects on H_2_O_2_ and 6-OHDA, indicating the presence of the activation of the SIRT1 enzymes. The toxicity of the TAT-α-Syn (A30P) and Aβ42 fibrils, both increased by ROS aggregation, was prevented by resveratrol. Resveratrol SIRT1 also lowered the toxicity of Aβ42, affected independent production and stability of Aβ42 fibrous, and reduced the production of intracellular Aß42-dependent ROS (Albani et al., 2009). Further research was done on the role of resveratrol in preventing oxidative stress in SK-N-BE, another cell neuroblastoma group. Sirtinol immune resveratrol with antioxidant effects on H_2_O_2_ and 6-OHDA shows a consequence of the activation of the SIRT1 enzyme. Resveratrol avoided the toxicity caused by TAT-α-syn (A30P) and Aβ42 fibrils, which increased the production of ROS. All fibrils were toxic. Resveratrol SirT1 also lowered the toxicity of Aβ42, impacted fibril development and stability independent of Aβ42, and decreased ROS generation dependent on Aβ42 (Vingtdeux et al., 2010).

### Resveratrol for Other Neurodegenerative Disorders: Therapeutic Agent

In addition to AD, for example, the neuroprotective activity of resveratrol mentioned above has shown Parkinson's disease (PD) and amyotrophic lateral sclerosis (ALS) to play a significant role. Both antioxidant and anti-inflammatory reactions aid resveratrol as well as SIRT1 and vintage activation, preventing oxygen stress deleterious effects. Resveratrol has recently been shown to be an excellent way of treating these neurological disorders. PD is a neuronal dopaminergic degeneration, which typically entails compacted and PD patients with increased muscle rigidity, restful tremor, bradykinesia, and, worst-case scenario, near-complete circulation loss. Ferretta et al. performed an *in vitro* experiment to find the effect of resveratrol treatment on primary fibroblast cultures from two patients with early-onset PD linked to different Park2 mutations. Resveratrol has demonstrated the regulation of energy homeostasis through the activation of AMPK and SIRT1 and increases the mitochondrial-oxidative function expression of several PGC1α target genes in the mRNA (Ferretta et al., 2014). Lin et al. used a model of PD caused by rotenone *in vitro* to check the neuroprotective effects of resveratrol. To verify this effect, Lin et al. used *in vitro* PD models induced with rotenone. Neuroprotection was regulated by resveratrol, which raises both HO-1 and autophagic flux without affecting cell viability. Consequently, the activities of the HO-1 drug inducer were close to those of resveratrol and healthy against death from rotenone cells (Lin et al., 2014). In the experiment in Khan et al., male rat Wistar has been used to establish a rat model of the pretreated, resveratrol-infected, 6-OHDA–mediated PD. Resveratrol has also been found to suppress COX-2 expression (Khan et al., 2010). Lofrumento et al. have continued investigating the neurosurgical effects of resveratrol on the PD model of 1.2.3,6-tetrahydropyridine (MPTP) from 1-methyl-4-phenyl. Results showed that resveratrol decreases glial, IL-1β, IL-6, TNF-α, and, respectively, its receptors in mice treated with MPTP SNpc (Lofrumento et al., 2014). Also, AMPK inhibition caused the elimination of the SIRT1 activity and decreased resveratrol protective effects for rotenone-induced apoptosis, which meant that the pathway of AMPK-SIRT1-autophagy in PD cell models played an important function in neuroprotection by resveratrol (Wu et al., 2011). Such findings show the effectiveness of resveratrol in PD diagnosis. ALS is a deadly adult neurodegenerative condition characterized by systemic cortex loss, brain stems, and motor neurons of the backbone. For the neuroprotective effects of resveratrol, a classic animal model of ALS was used, namely, the SOD1 transgenic mouse (G93A). Resveratrol has been found in this study to regulate the expression of Sirt1 and PGC1-α and inhibit P53 and its apoptotically downstream lipid peroxidation. Resveratrol delayed the onset of illness significantly, increased life span in the mouse, increased engine neuronal loss, and reduced atrophy and mitochondrial dysfunction. The antioxidant and anti-apoptotic activity of resveratrol is the principal advantage against ALS (Song et al., 2014). Additional work by Mancuso and others, including on resveratrol impact on ALS Mice SOD1 (G93A), has shown that the protective effects of resveratrol are linked to increased sirtuin 1, and the ventricular spinal cord expression of AMPK. Both mediators advocated self-flow standardization and, more specifically, improved SOD1(G93A) (Mancuso et al., 2014). The work of Wang et al. was proven similar to the upregulation of SIRT1, which protects SOD1-mediated toxicity from the ALS cell model (Wang et al., 2011). Both studies were a therapeutic target in ALS patients to prevent the degeneration of motor neurons.

### Poor Bioavailability of Resveratrol and Possible Solutions

We are also confused by its poor bioavailability, given the high bioactivity of resveratrol. Resveratrol plays an important role in AD, particularly *in vitro*, as we discussed earlier. However, when extended into *in vivo* animal and human clinical trials, it is difficult to demonstrate the same results. To date, the anti-inflammatory effects of resveratrol have provided different findings, including some observations that are inconsistent or controversial. Since resveratrol is poorly bioavailable, the level of resveratrol in the target tissues and cells is far from sufficient to prove efficacy in humans. The oral absorbance of resveratrol seemed to be at least 75%; however, due to the quick and extensive metabolism, biological availability was poor (Walle, 2011). Resveratrol has been rapidly transformed into sulfates and glucuronide metabolites in human liver and intestinal epithelial cells (Anekonda, 2006; Ponzo et al., 2014). *Trans*-resveratrol is photosensitive, easily oxidized, and undesirable for pharmacokinetics. The clinical effectiveness of resveratrol is therefore a big concern in drug products and medical technology. Scientists have been searching for analogs and designing new *trans-*resveratrol delivery mechanisms by coadministering the metabolism of *trans-*resveratrol inhibitors to improve solubility and bioavailability (Amiot et al., 2013).

Several studies in recent years have been based on new formulation techniques to stabilize and protect resveratrol from decay and improve its solubility to improve its bioavailability, ensure its continuous release, and ultimately transport resveratrol to some places through multi-particular shapes and colloidal carriers. Methylated resveratrol analogs conduct resveratrol-like biological activities. But as the methyl resveratrol analogs are easier to transport and are more resistant to degradation, greater bioavailability is demonstrated. First, Kang and others documented an artificial biosynthesis route to MRC in *E. coli* culture and two genes for O-methyltransferase along with biosynthetic genes of resveratrol (Kang et al., 2014). Friend et al. developed novel galenic-soluble powder form (40 mg) which was dissolved in a complex mixture of 20 polysorbate and 3-dioleate polyglycerol. Consequently, *trans-*resveratrol absorption and complete bioavailability of *trans-*resveratrol (+780%) were significantly improved by the novel formulation. In two lipid caplets (40 mg), a single dose of soluble *trans-*resveratrol may increase *trans-*resveratrol plasma concentrations by 10 times to dry powder type (Amiot et al., 2013). Amri et al. used a monodisperse to operate a porous polymer microsphere to stabilize and maintain resveratrol (Amri et al., 2012). On the other hand, the formulas for achieving a predicted or continuous release of resveratrol were multi-particular shapes in micrometer or colloidal nanometer carriers in specific experiments. Das et al. discovered that pectinate beads (>97.5%) and Zn pectinate beads can be used to delay releases that provide the lower gastrointestinal tract with specificity on site (Das et al., 2010; Frozza et al., 2010a and 2010b). It may be a good idea to monitor the release and improve the bioavailability of resveratrol microparticulate systems. Researchers have recently injected resveratrol into the interconnected vanillin chitosan microphones to facilitate stability, and the resveratrol encapsulation effectiveness in microphones was 93.68% (Peng et al., 2010). In another test, pectin-hardening agents have been developed to connect pectin molecules to zinc ions and glutaraldehyde, with >94% encapsulated efficiencies within the colon (Das and Sahoo, 2011). In pharmaceutical applications, cyclodextrins that solve and bind to form polymers are used to supply drugs with hydrophobic drugs. The bioavailability of poorly soluble drugs can be improved, like resveratrol, and the water solubility can be improved by transforming crystal cyclodextrins into amorphous mixtures of isomeric derivatives. Resveratrol was tested in people in an enhanced antioxidant effect as opposed to a *trans-*resveratrol formulation only by β-cyclodextrin-[βCD-] formulation care (Moyano-Mendez et al., 2014). The drug's solubility has been enhanced, and the overall bioavailability of resveratrol nanosuspensions has increased with high pressure and stable nanosuspensions (Kobierski et al., 2009). Solid lipid nanoparticles (SLNs) can be used as an alternative carrier to resveratrol in several studies. Resveratrol solubility, stability, and intracellular loading into SLNs have all been improved (Teskač and Kristl, 2010). The Jose et al. study shows resveratrol-loaded SLNs as well as free resveratrol and SLNs as opposed to free resveratrol may substantially increase the brain concentration for resveratrol. The findings have thus shown that the SLN loaded with resveratrol is a promising therapeutic device in brain tissue (Moyano-Mendez et al., 2014).

A vesicular system, which means colloidal particles and ethosome-concentrated two-plate, which are capable of carrying hydrophilic as well as hydrophobic medicines, is another novel way of providing encapsulated drugs. Vesicular systems can boost bioavailability and have longer term therapeutic activities, including liposomes, transfers, and phytosomes. The formulation of liposomes was selected in a study to optimize the burden of rigid resveratrol and liposome and to improve the bioavailability of pharmaceutical products (Bonechi et al., 2012). Different surfactants and lipids have been used and characterized in terms of thickness, zeta potential, stability, and permeation for the preparation of transferases and ethosome. For preparing resveratrol-laden niosomes, Pangeni et al. used Span 60 and Span 80. This made the latter more stable with a narrow particle distribution and a high trapping efficiency, which demonstrated improved bioavailability (Pangeni et al., 2014). Nanosponges are nanohorizons in nanotechnology for the delivery of drugs. They can be mixed with poorly soluble resveratrol in the substance and increase bioavailability due to their small size and porous nature (Ahmed et al., 2013). Resveratrol encapsulations in β-CD nanosponges enhanced their water solubility, and researchers found resveratrol-charged nanosponges feasible for oral and topical delivery systems (Ansari et al., 2010). A lipid-based resveratrol delivery system with the ability to self nanaemulsify Acrysol K 150 as a lipid and Transcutol HP as a surfactant was developed recently by Pund and colleagues. This improved lipid-based nano-emulsifying resveratrol *in vitro* solubility and *in vitro* efficiency (Pund et al., 2014).

### Resveratrol Clinical Trials in AD

Resveratrol can benefit *in vitro* and *in vivo* assays in the treatment of AD. There is growing evidence. However, until now there has not been a complete large-scale clinical trial; resveratrol is well absorbed, and toxicity is not reported significantly. Resveratrol is not bioavailable, has low water solubility, and chemically unstable because of its poor bioavailability (Walle, 2011). Resveratrol was easily metabolized in humans, including a high within 30 min, within 2 h of oral or intravenous injection of resveratrol (Anekonda, 2006). A number of *in vivo* experiments in animals and humans showed very poor intestinal absorption, and the identification of unmetabolized resveratrol in the circulating plasma was difficult (Cottart et al., 2010). Upon oral administration of 200 mg three times a day, trans-resveratrol pharmacokinetics was shown to be independent of age. Nevertheless, trans-resveratrol plasma rates were fairly small (Nunes et al., 2009). Although there is *in vivo* evidence that resveratrol is bioavailable and bioactive in animal models, definitive results in human trials remain lacking. Several researchers have sought in recent years to find the best ways to treat resveratrol (Amiot et al., 2013). Some work has demonstrated the biological resveratrol metabolites and analogs with identical neuroprotective properties (Saiko et al., 2008). Numerous clinical trials are underway to analyze the effect of resveratrol on neurological diseases, including AD, despite the enormous challenges (Kennedy et al., 2010). The overall concentrations of hemoglobin in the cortex increased significantly during job success in a two-blind cross-checked study of healthy young volunteers who ingested 500 mg of resveratrol (Patel et al., 2011). However, it had no impact on cognitive performance. As a randomized controlled trial (RCT) placebo dietary supplement, 60 patients received a fluid glucose resveratrol/maleate. Up to 1 year after the start of the analysis, the ASD-Cog values were regularly monitored (Mecocci and Polidori, 2012). The ADAS-Cog rating was calculated for six-month period in another multi-intervention trial with mild cognitive impairment, resveratrol, or the caloric limitation or omega-three or placebo addition (Xu et al., 2015). Resveratrol analogs which show better bioavailability, efficacy, and stability than Res are being tested for their activity in relation to many degenerative conditions. Some studies showed that Res derivatives exhibit neuroprotective effects in *in vivo* and *in vitro* models (Kim et al., 2007b).

## Concluding Remarks

Resveratrol (3,4,5-trihydroxystilbene) is a polyphenolic naturally occurring compound that is found in more than 70 species of plants, herbs, or human food material like berries, grapes, and peanuts. It is also beneficial to cure several other diseases like cardiovascular disease, pain, cancer, tissue injury, and inflammation, and also used in AD. Due to its multiple neuroprotection mechanisms, resveratrol is a new agent for treating AD. Because the treatment of AD remains a global issue, resveratrol therapeutic potential has attracted the researchers' attention. Many studies have been performed in cell culture and in the animal model of AD to find precise data on the neuroprotective mechanisms of resveratrol. Resveratrol also has beneficial effects on AD for antioxidant and anti-inflammatory purposes. However, official clinical studies have also been conducted on resveratrol care in AD. While the problems of clinical applications such as bioavailability, dosage, and side effects are enormous, scientists still seek to investigate the comprehensive mechanism and the effective clinical administration of resveratrol.

## Author Contributions

MA‐D and MHR conceived the original idea and designed the outlines of the study. MHR, RA, TB, SA, MA, and NA‐J wrote the draft of the manuscript. MHR, NA, and AA prepared the figures and tables of the manuscript. MHR, OA‐E, HE‐S, DK, VM, and MA‐D performed the literature review and improved the manuscript. All authors contributed to the article and approved.

## Funding

The authors extend their appreciation to the Deputyship for Research & Innovation, “Ministry of Education” in Saudi Arabia for funding this research work through the project number IFKSURP-99

## Conflict of Interest

The authors declare that the research was conducted in the absence of any commercial or financial relationships that could be construed as a potential conflict of interest.
